# “You can’t just eat 16 teaspoons of sugar so why would you drink 16 teaspoons’ worth of sugar?”: a qualitative study of young adults’ reactions to sugary drink warning labels

**DOI:** 10.1186/s12889-022-13648-1

**Published:** 2022-06-22

**Authors:** C Miller, K Wright, J Dono, S Pettigrew, M Wakefield, J Coveney, G Wittert, D Roder, S Durkin, J Martin, K Ettridge

**Affiliations:** 1 The University of Adelaide’s School of Public Health, Adelaide, Australia; 2 Health Policy Centre, South Australian Health and Medical Research Institute, North Terrace, Adelaide, South Australia 5000 Australia; 3 The University of Adelaide’s School of Psychology, Adelaide, Australia; 4 Food Policy, The George Institute for Global Health, University of New South Wales, Sydney, Australia; 5 Centre for Behavioural Research in Cancer, Cancer Council Victoria, Melbourne, Australia; 6 School of Psychological Sciences, The University of Melbourne, Melbourne, Australia; 7 College of Nursing and Health Sciences, Flinders University, Adelaide, Australia; 8 Freemasons Foundation Centre for Men’s Health, Faculty of Health Sciences, University of Adelaide, Adelaide, Australia; 9 Centre for Nutrition and GI Diseases, South Australian Health and Medical Research Institute, Adelaide, Australia; 10 Cancer Epidemiology and Population Health, University of South Australia, Adelaide, Australia; 11 Obesity Policy Coalition and Alcohol and Obesity Policy, Cancer Council Victoria, Melbourne, Australia

**Keywords:** Young adults, Sugary drinks, Warning labels, Qualitative, Consumer perceptions

## Abstract

**Background:**

Several jurisdictions have introduced nutrient warning front of pack (FoP) labels in an effort to curb consumption of ultra-processed foods and beverages high in free sugars (sugars added to foods and beverages, and sugars naturally present in honey, syrups, fruit juices and fruit juice concentrates). This study aimed to explore consumer understanding and perceptions of FoP warning labels that convey different nutritional and health information messages regarding the consumption of sugary drinks.

**Methods:**

Sixteen focus groups were held with 4–8 young adults per group (aged 18–24; *n* = 105 participants in total) stratified by education level, location (rural centres, large cities) and gender (males, females) to ensure diversity. Labels shown to participants during group discussions included text warning labels of health effects, exercise equivalents, calorie/kilojoule information and sugar content as a “high in” label and as teaspoons (text and pictograms). Thematic analysis was undertaken.

**Results:**

Four themes were identified related to participants’ perceived effectiveness of labels: the extent to which labels were perceived to be useful, relevant and credible; the extent to which a label elicited shock or disgust (perceived aversiveness); the extent to which the label message was resistant to self-exemption; and participants’ perceived potential of the label to reduce purchasing and consumption behaviour. Across all four themes, labels communicating the number of teaspoons of sugar in a sugary drink (whether by text or pictogram) were perceived as the most impactful, resistant to self-exemption and to have the greatest potential to reduce consumption, with enhanced reactions to the pictogram label. Labels depicting health effects, exercise equivalents, calorie/kilojoule information or a general ‘high in sugar’ warning were perceived by consumers to be less effective in one or more themes.

**Conclusions:**

Labels conveying the amount of sugar in a beverage in teaspoons were perceived as highly factual, relatable and interpretable, and as having the greatest potential to impact consumption attitudes and intentions. Further quantitative studies are required to compare the potential effectiveness of the teaspoons of sugar labels in reducing purchasing and consumption behaviour than other alternative warning labels, such as health effects or “high in” sugar labels.

**Supplementary Information:**

The online version contains supplementary material available at 10.1186/s12889-022-13648-1.

## Background

Sugar-sweetened beverage (SSB) consumption has become a focus of global public health efforts, as excess SSB consumption is causally associated with increased risk of obesity, dental caries, type 2 diabetes and cardiovascular disease risk factors [1–4]. SSBs are low in nutritional value and high in free sugars [5].The World Health Organization (WHO) defines free sugars as including “monosaccharides and disaccharides added to foods and beverages by the manufacturer, cook or consumer, and sugars naturally present in honey, syrups, fruit juices and fruit juice concentrates” [6]. The WHO has recommended that free sugars be reduced to less than 10% of total energy intake, with a further conditional recommendation that reducing free sugars to less than 5% of total energy intake would provide additional health benefits [6]. Just over half (52%) of all Australians exceed the WHO’s recommended free sugar intake, and nine out of ten exceed the conditional recommendation [7].

Beverages are the largest contributor to free sugars in the Australian diet (52% of total free sugars), of which soft drinks, sports drinks and energy drinks comprise the largest sub-category (19% of free sugars), followed by fruit drinks (13%), sugar added to tea and coffee (7%) and cordials (5%) [7]. Australian adults aged 18–24 years are the highest adult consumers of SSBs, with 61% of young adults consuming SSBs at least once per week and 14% consuming SSBs daily. A recent national Australian study has demonstrated deficits in knowledge of the nutritional composition and health effects of SSBs [8]. Public health interventions to increase awareness of potential negative health effects of SSB consumption and promoting healthier consumption behaviours are needed [9–11].

Warning labels on front of pack (FoP) for SSBs have been proposed as a policy to assist consumers in making an informed choice at the point of purchase or point of consumption. More than 30 countries around the world have passed or implemented FoP labels, including Chile, Ecuador, Iran, Sri Lanka, Mexico, Peru, Israel and Uruguay, with Chile being the first country in 2016 to implement “high in…” nutrient warning labels to alert consumers to excessive amounts of harmful nutrients, such as sugar [12–14]. In Australia, warning labels on SSBs have not been implemented to date, despite high levels of public support [15] and a growing body of evidence demonstrating their effectiveness in reducing the consumption of sugary drinks [16, 17]. A meta-analysis of 23 studies demonstrated that warnings on sugary drinks can significantly reduce purchasing and consumption outcomes (hypothetical and actual) [16]. Sugary drinks warning labels were more noticeable, yielded stronger negative emotional reactions (e.g., worry, disgust) and prompted consideration of health effects or harms of consumption compared to control or no label conditions. The labels included in the studies in this meta-analysis generally depicted either health effects or nutrient warnings, with some difference observed in outcomes according to these label types across studies. While both types of labels, that is, those referring to health effects (e.g., obesity, diabetes) and those referring to nutrient warnings (e.g., “high in” or “excess” sugar) resulted in a reduction in hypothetical purchasing, the labels warning of adverse health outcomes were found to have stronger effects in this meta-analysis. A scoping review of 22 studies [8 of which were included in the study cited above [16]] compared nutrient warning labels (e.g., a product contains high levels of nutrients of concern such as sugar, fat or artificial sweetener) on SSBs against other warning label systems [17]. The review found that FoP nutrient warnings attracted attention, were easy to understand, helped consumers identify unhealthy products and discouraged consumers from purchasing unhealthy products. However, other labelling systems such as health effects warnings convey more information and enabled consumers to gain an overall sense of the relative healthiness of products. These review papers demonstrate the nuances in labelling that may differentially impact motivation to reduce consumption and actual behaviour.

While these experimental studies provide strong quantitative evidence of the potential effectiveness of SSB warning labels, few studies have explored consumers’ underlying reasoning for the effectiveness of different warning labels applied specifically to SSBs. To date, one US qualitative study which included semi-structured interviews of 25 women (pregnant women and/or mothers of infants/toddlers) and seven healthcare providers found that SSB warning labels that included messages regarding the health consequences of sugary drink consumption or illustrations of the sugar content were viewed as highly acceptable, and sugar content information was useful to inform decisions [18]. Other qualitative studies conducted in South American countries have reported on consumers’ perceptions of FoP warning or nutrition labels, or health messages more broadly, for foods and/or drinks high in salt, sugar or fat [19–23]. A study of Chilean mothers’ perceptions of labelling law found that warning labels were perceived to be influencing children to make healthier food choices [22]. A study of Brazilian adults found that key barriers to participants using food labels were that they lacked the time, requisite knowledge and skills to interpret complex nutrition information labels, and therefore felt that FoP warnings written in everyday language would be preferred [21]. Comparisons between these studies are limited due to variation in label types, health messages conveyed and participant groups. However, overall, there was a general indication of acceptability of warning labels applied to food products, including SSBs, and the perceived potential for warning labels to improve knowledge and change behaviour. Findings also suggested that simple information written in plain language was preferred. While such findings are insightful, further qualitative studies providing in depth exploration regarding “why” certain warning labels are (or are not) likely to be effective in reducing SSB consumption, or reasons underlying differences in acceptability and understanding, are still lacking.

Consumer perceptions and understanding of nutritional information and warning labels are likely to vary with context. To date, qualitative explorations have most commonly been conducted in South American countries. With many countries and jurisdictions yet to mandate any FoP warning labels for SSBs, further insight is needed to facilitate the development and implementation of such labelling approaches. This information would provide policy makers with further insight into consumers’ reactions to health warning labels which contribute to their political feasibility as an intervention to reduce consumption. As noted, young adults have the highest SSB consumption among Australian adults, therefore, they would be one of the groups most impacted by policy interventions. The current study aimed to explore among young adults, consumer understanding and perceptions of health warning labels that conveyed different health information messages regarding the consumption of sugary drinks. The health information warning labels pertained to either nutritional (sugar, calories, kilojoules) content, health effects information (obesity, diabetes, tooth decay) or exercise equivalents (that is, the time it would take either running or walking to eliminate the calories consumed), based on previous literature and labelling systems [12, 24, 25]. Specifically, we explored the extent to which the labels provided new knowledge and motivation to reduce sugary drinks consumption and the reasons underpinning these perceptions.

## Methods

### Design

A qualitative descriptive approach was employed for this study [26, 27]. The study was approved by the Human Research Ethics Committee of the University of Adelaide. MMresearch, a social research company based in Melbourne, Victoria, was commissioned to recruit participants and run the focus groups. Procuring an external person to run and moderate the focus groups can improve confirmability such that findings reflect participants’ responses and not the bias of the researcher or research group [26, 28]. Sixteen focus groups were conducted with adults aged 18–24 years. Groups were stratified by location (Adelaide, Perth, Sydney, and Ballarat (rural Victoria) – 4 groups per location); gender (50% female); and level of education (50% tertiary, 50% no tertiary education) to elicit a diversity of responses rather than ensure population representativeness or compare responses between these attributes. These attributes were prioritised for stratification for this research as our previous national survey of Australian adults indicated soft drink consumption and knowledge and beliefs related to soft drink consumption varied according to these factors, and therefore, responses to labels may vary with these attributes [8, 29]. We also wanted to ensure we captured male responses given they are the highest consumers and are often under-represented in health research [30]. Locations were selected across Australia to include larger, smaller and regional cities/areas. A maximum of 8 participants per group was set, with a total potential maximum of *n* = 128.

### Participants

MMresearch coordinated participant recruitment through professional recruitment companies based within each state from their existing participant pool. Initial contact was an email containing information regarding the study. Those who expressed interest were interviewed by phone to check eligibility. Participants were eligible if they were aged 18–24 years, consumed sugary drinks at least weekly (defined as soft drinks, sports drinks and/or energy drinks that contain added sugar, i.e., not sugar-free, and not including milk, tea, coffee, fruit juice, artificially sweetened drinks or alcohol-based drinks), they or close friends/family did not work for the beverage marketing or manufacturing industry and provided informed consent. Stratification was tracked during recruitment. Where a maximum quota for a location, gender and/or education level was already reached, further participants were excluded from that strata. The total number of participants was *n* = 105, aged 18–24 years, across 16 groups. See Table 1 for participant numbers and composition for each group. Groups were conducted face to face and run separately according to gender and education at each location.

**Table 1:** Number of participants by location, education level and gender (*n* = 105)

	Lower Education level		Medium–High Education level	
Location	Male	Female	Male	Female
Adelaide	7	5	8	8
Ballarat	6	7	4	8
Sydney	6	6	8	7
Perth	6	7	6	6

### Procedure

**Fig. 1:**
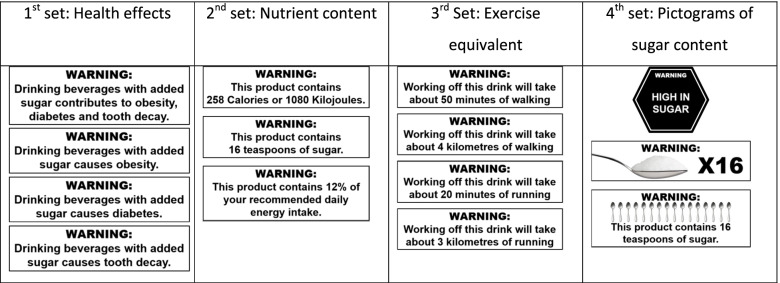
Warning labels as shown to participants, with each label from within a set shown individually, then as a full set

MMresearch, a social research company based in Melbourne, Victoria, was commissioned to moderate the groups using a semi-structured discussion guide (See supplementary material). The discussion guide was developed in line with previous studies in tobacco and sugary drinks to assess perceived effectiveness of labels using a set of commonly used cognitive indicators [17, 31]. The 14 warning labels used in this study were adapted from examples of labels tested within the quantitative SSB warning label literature [24, 25], materials used in studies on strategies to reduce SSB consumption [32, 33] and the real-world label used in Chile [12] (see Fig. 1). Groups were face-to-face, and participants were informed that the focus group’s purpose was to explore people’s attitudes, knowledge and behaviour in relation to sweet drinks. They were advised that the groups would be audio and/or video recorded to allow for transcription and that no individually identifying information would be reported or published. Following the initial warm-up discussion, participants were exposed to 14 warning labels covering four categories: health effects (4 labels), nutrient content (3 labels), exercise equivalent information (4 labels) and pictograms of sugar content (3 labels). When creating labels, a 600 ml Coke was used as a reference point to calculate quantities as it was the most commonly consumed sugar-sweetened beverage in Australia. Time was allocated to discussing ready to consume beverages at the beginning of the focus groups to ensure participants were familiar with drinks that were relevant to the labels in the study. Labels were presented as a slide show on a projector screen in a set sequence where labels within a category were first presented individually and then alongside the other labels within the same category (e.g., each heath effect label was shown individually then shown together as a set before moving onto nutrient content labels). After viewing an individual label, participants were asked open-ended questions about their initial perceptions and whether it was attention grabbing, provided new information, was believable, was relevant and/or prompted reconsideration of their current sugary drink consumption. The moderator used iterative questioning and did not impose views on participants. After viewing a set of labels within the same category, participants were asked which stood out the most in that category. Data saturation was concluded by the research team in conjunction with the moderator through ongoing iterative discussions of findings arising from the groups, such that by conclusion of the final focus group there were no new concepts arising. Focus groups were conducted in 2017 and were approximately 90 min in length. All 12 groups from larger metropolitan cities (Perth, Adelaide, Sydney) were video-recorded, with the four groups conducted in the regional area of Ballarat audio-recorded due to lack of video-recording facilities. Participants were reimbursed $80 AUD for their time.

### Analyses

Thematic analysis was undertaken following the approach of Braun and Clarke for data engagement, coding and theme development [34]. The core research team (CM, KE, JD, and KW) viewed or listened to the focus groups either live or via recordings. One researcher immersed themselves in all recordings as well as the de-identified transcripts. This researcher coded the data using NVivo software [35] and used both inductive and deductive coding based on a simple framework developed among the core research team from their observations of the groups and the discussion guide. This coding was first done within each label set, then the researcher identified common themes across the label sets. The researcher employed a reflexive approach during this phase of the analysis such that they continually re-checked between the data and their developing themes. Themes and coding frames were continually discussed among the core research team which served as a validity check of the interpretation of the data, leading to the final set of themes that was agreed upon within this team. Quotes from participants are presented with the following group descriptors: group number (G), group gender (male or female), group education level (low (Low) or medium–high (M-H)) and group location (Adelaide, Ballarat, Sydney or Perth). Where an excerpt of conversation exchange is reported between one of more participants, a participant number is used to denote when a new person is talking, e.g., P1 and P2 denote Participant 1 and Participant 2.

## Results

Four common themes were identified. These are summarised in Table 2 and discussed in detail below. In summary, the teaspoon labels (whether text or pictogram) were a clear standout, as they appeared to rapidly increase participants’ knowledge of the sugar content of a beverage and prompt serious reconsideration of consumption of sugary drinks. Responses to all other labels in this study were varied in terms of perceived potential effectiveness.

**Table 2:** Summary of themes

Theme	Description
Prerequisites to effectiveness	Participants believed that labels had to be credible, personally relevant and contain useful information to capture interest and attention.
Perceived aversiveness	Participants reacted to the perceived aversiveness of the label information, this was regarded as the extent to which a label elicited a negative emotional response (e.g., worry/concern, disgust). For some labels this was negligible, while others induced strong reactions. Perceptions of aversiveness appeared to be influenced by personal experience.
Self-exemption	For most labels (including those that participants considered serious in nature, e.g., increased risk of diabetes), participants could provide a self-exempting rationale.
Perceived potential to reduce personal sugary drinks consumption	Participants appraised the potential of the label to produce real change in their motivation to reduce consumption, for example, it may: prompt a participant to reconsider an SSB purchase; motivate reduced SSB consumption; encourage switching to a different product; or not motivate any change. This theme was strongly related to the aforementioned themes such that a label perceived strongly across all other themes was likely to be perceived to have potential to reduce consumption, however, a label that was perceived strongly on only one or two themes (such as only prerequisites to effectiveness) would not necessarily be appraised by participants to have real potential to reduce consumption.

### Prerequisites to effectiveness

Overall, comments indicated that warning labels perceived as lacking credibility would likely result in the information being rejected. Several factors appeared to influence perceptions of credibility. For example, the word “warning” was described as indicating that the label contained serious information, and for some, perceptions of seriousness arose from drawing a parallel with the health warning labels displayed on cigarette packaging. Conversely, some participants asserted that warning labels “didn’t work” for cigarette smokers, therefore it would be pointless putting these labels on sugary drinks.

The health effects labels were easily understood and perceived as a useful reminder of the health risks by most participants. However, some participants indicated the obesity information was obvious and not of interest. The word “causes” in the health effects warning labels stimulated discussion among participants, including comparing this wording with “contributes to”. Overall, “contributes to” was perceived to be more realistic and credible by many participants, though there was acknowledgement by some that this wording could weaken the health message. Some participants objected to the word “causes”, perceiving it to be being inaccurate, particularly regarding the obesity label. They argued that the wording on the label was “not true” because “other things cause obesity too” (such as junk/sugary/fatty foods) and/or the effects of obesity were not an immediate or guaranteed outcome.

“Well, that’s not true. That’s not necessarily true, right? Like, if I drink one of those Cokes right now, I’m not going to become morbidly obese tomorrow.” [G10 Male M-H Sydney]

“And, it’s better that it says contributes rather than causes…Because everything causes everything else. But it.. like it’s not one thing that’s going to cause you to get diabetes. If you drink soft drink all your life, it’s probably not the only thing that’s going to cause it.” [G05 Female M-H Ballarat].

The links between sugar and both diabetes and tooth decay were generally well-known and accepted, and therefore credibility of this health effect was higher. Credibility was not questioned when participants were presented with factual information about exercise, sugar and calories. However, perceptions of relevance of the information did vary according to their existing knowledge and experiences. Exercise information (both distance and minutes) provided interesting and useful information to participants who exercised regularly, as it communicated knowledge about how to “burn off” a sugary drink. For others, minutes were easier to relate to because of difficulties visualising distances.

Responses to the content labels highlighted that participants needed to be able to understand the information in order to relate to it. The words “calories” and “kilojoules” were familiar; however, understanding of these terms, along with “recommended daily intake” and “energy”, was very limited. Many participants commented that they needed more information as context for “258 cal or 1080 kilojoules” and “% of your recommended daily energy intake”. Some participants commented that 258 cal and 12% seemed like small quantities, and therefore SSBs are acceptable to drink. Only a small number of participants recognised that the exercise requirements would be additional to their current level of physical activity.

“It’s, to me it’s digits. Unless you really know what it actually means. There’s no point, you know.” [G08 Female Low Ballarat] (in response to the calories/kilojoules label)

“The word ‘energy’ confuses me. I don’t really understand what energy is measured in, if it’s kJ or… like, I don’t understand what energy… it’s sugar or…” [G01 Male M-H Adelaide]

“That one wouldn’t probably fuss me so much ‘cos I’d look at it be like well I’ve still got 88% to go, so…” [G04 Female Low Adelaide] (in response to the 12% Recommended Daily Intake [RDI] energy label)

“There’s still 88% left.” [G05 Female M-H Ballarat]

In contrast to the other content labels, the teaspoons of sugar labels (whether text or pictogram) were perceived to provide information that was credible, relatable and useful. Note that the teaspoons text-only label (set 2) was shown prior to the pictogram versions (set 4). In response to the text-only label, teaspoons were perceived as a familiar measure that is easy to visualise. The pictogram versions provided even clearer visualisation of the quantum of sugar that would be consumed from the beverage. In particular, the pictogram label showing an image of one teaspoon of sugar with “ × 16” was considered to enhance the message. Participants commented that teaspoons labels would allow for easy comparisons between different beverages and/or to add up the number of teaspoons consumed across the day/week, enabling more informed choices. Some commented that they preferred teaspoons because it was a simple message compared to the other labels they were shown.

“I suppose it’s a realisation too. Like you can have your numbers of your calories and whatever and you can have ‘it may cause diabetes or it can contribute it’, but you see, like you would use a teaspoon every day, so you would see how many 16 teaspoons are going in and that would make you stop and think that, that is a lot of sugar.” [G08 Female Low Ballarat]

“And I guess what I’m thinking is, the middle one, the ‘warning × 16’, it would be a pretty easy way to compare if you’ve got five different drinks, and you are thinking out of this, this or this, and this one says 13, this one says 16, this one says 14.” [G06 Male Low Ballarat]

Conversely, the “High in sugar” pictogram label was easily understood, but so vague it would not be useful if broadly applied. Having an indication of the sugar content was considered better to enable a healthier (lower sugar) choice rather than simply labelling all beverages as high in sugar.

“It doesn’t have meaning. There needs to be a link to the consequences, like before – the diabetes, the obesity, or something. But ‘high in sugar’? Yeah, so what?” [G01 Male M-H Adelaide]

“I think if I saw a big cabinet full with all the drinks in there saying that, well, I can’t win. I can’t choose anything without having that, so I’m still going to choose one because there’s no option.” [G05 Female M-H Ballarat]

### Perceived aversiveness

Participants had varied degrees of emotional responses to the labels in terms of the extent to which they found labels aversive (caused worry or disgust). For some, there was no emotional response elicited such that label information was quickly dismissed as neutral or of negligible importance because it was reportedly well-known (e.g., obesity) or too abstract (e.g., calories or kilojoule disclosures). However, for other labels (such as the teaspoons labels), emotional responses were more prominent. Emotional responses were regarded as participants’ expressions of intense dislike verbally and/or through facial expressions of disgust and worry when discussing what the label made them visualise or feel.

All three health effects were perceived as undesirable (i.e., aversive), albeit not to the extent that they would reduce consumption for many participants. There were negative perceptions of obesity and tooth decay arising from perceptions of stigma relating to how these health conditions may change personal physical appearance. For tooth decay there was also talk of the discomfort and expense of visits to the dentist. By contrast to obesity, diabetes was perceived to be silent in onset (no visible signals of disease development) and irreversible, and responses indicated that it was feared by many. Knowing someone who had experienced a particular health effect increased the personal relevance and perceived aversiveness for some participants.

“I think (diabetes) is more worrisome because you don’t have to be overweight to have diabetes and with being overweight, you go, hey, look, I’m gaining a kilo, I’m gaining another kilo. You can be prepared for it. Diabetes – it’s just, oh, shit, the doctor says I’m, like, f-ed.” [G11 Female Low Sydney]

The perceived aversiveness of the information provided in the exercise labels varied according to the individual participant’s perception of the degree of effort that would be required (and was not related to reported current physical activity levels). There was no consistent pattern regarding which specific exercise message (in minutes or kilometres) was more aversive, this appeared to differ between individuals.

The calorie/kilojoule information was only aversive to participants with personal experience using this information previously, e.g., through dieting behaviour. By contrast, several participants commented that energy (viewed on the energy label) was considered a positive attribute, e.g., providing an energy boost.

Excerpt of exchange between two participants in the same group:

P1:“I’d be like, I need more than 100% of my daily energy anyway, so, like, I’ll just have extra.”

P3:“My energy’s too low, I need more.”

[G05 Female M-H Ballarat]

Overwhelmingly, the teaspoons of sugar labels (whether text or pictogram) elicited responses of shock and disgust. With respect to the text teaspoons label, many participants commented that they would not consume that many teaspoons of sugar (e.g., eating it in their food or drinking it in their tea or coffee) and found the thought physically repulsive.

“You can’t just eat 16 teaspoons of sugar so why would you drink 16 teaspoons’ worth of sugar?” [G04 Female Low Adelaide]

“I think the other ones, you have to think about them too much, but 16 teaspoons of sugar, it’s like, ‘Ok, yup, I get it, that’s a lot’.” [G01 Male M-H Adelaide]

The pictogram teaspoon labels elicited particularly strong reactions with the label prompting specific mental imagery of the total quantum of sugar consumed for some participants. Some participants commented that such a high sugar content seems excessive or unreasonable, or even ‘scary’ in terms of health effects.

“Uh. That is so gross. Reminds me of a cigarette packet. Like it’s, it can do scary things.” [G11 Female Low Sydney]

“You visualise and can imagine doing it straight away and you go, ‘Nah, that’s disgusting’.” [G01 Male M-H Adelaide]

“It’s like a heart attack.” [G08 Female Low Ballarat]

Excerpt of exchange between two participants in the same group:

P4:“I’m thinking about my Weet-bix at home, like in the morning when I put sugar on my Weet-bix like 16 teaspoons on to food.”

P5:“Yeah I wouldn’t put it on food. Why would I put it in my drink?”

[G12 Female M-H Sydney]

### Self-exemption

Many participants were adept at self-exempting from the information conveyed by most labels, even those perceived as highly aversive, for example, those stimulating thoughts of insulin injection (diabetes label) or requiring high physical effort (exercise equivalent). Some self-exempted via appraising the threat to be not imminent (e.g., diabetes/obesity occur in older people), deflecting the risk or engaging in remediating action (e.g., brushing teeth mitigates risk of tooth decay or seeing weight gain occur and acting to prevent or reverse obesity). Many participants indicated they already engaged in the required level of physical activity to offset beverage intake, that they could easily adjust their diet to accommodate consuming a drink or that their metabolism would naturally “burn it off”.

The teaspoons labels were a notable exception; participants appeared to find it difficult to self-exempt from the information due to the meaning being quickly and easily understood (as a teaspoon is a relatable measure, easy to visualise and doesn’t need contextualising) and their pre-existing knowledge that too much sugar is “bad”. Again, the effects of the pictogram labels were especially strong.

“For me it’s like calories … you can have like 1,600, so when I see that … I think, okay I can have this to contribute to the 1,600. Whereas 16 teaspoons of sugar, I’m like, I don’t want that in my body.” [G12 Female M-H Sydney]

### Perceived potential to reduce sugary drinks consumption

Participants perceived the health effects labels as having limited potential to impact motivation to reduce sugary drinks consumption. The diabetes and tooth decay labels appeared to have slightly greater potential effect than the obesity label. A small number of participants said that they would re-think their sugary drink consumption, but most said that exposure to the labels would not change their behaviour.

For some participants, the exercise information labels prompted thoughts of earning the beverage through exercise, rather than prompting them not to drink it. For the majority, however, it appeared this information, while considered interesting, was perceived as being unlikely to change their own behaviour. A small number of participants who were already highly literate in the measures indicated that the calories/kilojoules label would reduce their consumption, but for everyone else the label was considered too abstract or meaningless to prompt behavioural change. The 12% RDI label was perceived by participants as though it could possibly promote consumption (both their own and/or others’), given that energy could be perceived as a benefit and 12% was perceived as a small amount. Due to the lack of specificity, the high in sugar pictogram label was perceived to have limited potential to motivate behaviour change.

“That is less impactful for me, because it’s like, I can have ten of these and be fine.” [G15 Male M-H Perth] (referring to the 12% RDI energy label)

By comparison, the teaspoons labels (text and pictogram) were described as having good potential to motivate a reduction in SSB consumption. Many participants indicated that exposure to these labels was likely to motivate them to switch to a lower sugar beverage, consume less, not consume the beverage, or at least make them think twice about their decision to consume. The following responses were in response to the text teaspoons label:

“Yeah, even like if you did know about it [Calories, energy intake] – because I know a little bit about it, managing calories and whatnot – but still it’s not as easy to understand. Like, you know, you’ve got to sit there a little bit and go, that’s like how many in regards to this type of thing. Whereas 16 teaspoons – that’s a lot.” [G01 Male M-H Adelaide]

“I think it’s good in some regards because someone can visualise how many teaspoons you put in a cup of coffee or something. It’s a very basic and primitive figure almost. But you’re not knowing the exact amount obviously, you know. A teaspoon here can be different to a teaspoon there. But I think it’s better for those who are less educated or aren’t as likely to go look at the back of the packaging.” [G02 Male Low Adelaide]

The pictogram teaspoons labels appeared to enhance the reaction, and specifically, the image of one teaspoon of sugar with “ × 16” seemed to prompt participants to rethink their consumption and purchasing behaviour.

“It would definitely stop me from drinking it… if there’s, 16 teaspoons are going into my system right now, I’m like, just pick up the water bottle instead.” [G11 Female Low Sydney]

“Yeah, the ‘16’ being massive on that one too. You look at it and go ‘16 teaspoons of sugar’ instantly. The bottom one [image of 16 individual teaspoons], you’ve got to look at it and read and go, ‘Oh, 16’.” [G01 Male M-H Adelaide]

“It would definitely make you think twice like, even after you’ve finished the drink, and it’s in your car, like the empty bottle, you’d look at it… and then you’d think about well, 16 teaspoons for what?” [G09 Male Low Sydney]

Excerpt of exchange between three participants in one group:

P2:“Yeah. I think it would turn off a lot of people, to be honest. Yeah.”

P6:“If I walked past that, I’d look at it and I’d go, that makes me feel sick. I’m not going to get any soft drink any more like.”

P5:“You would think about the bottle and think about all that sugar floating in it, and, literally, be like, nah, sugar.”

[G14 Female Low Perth]

## Discussion

This study explored young adults’ responses to different potential warning labels including the underlying reasons regarding why (or why not) labels were perceived as having potential to change knowledge and/or reduce consumption of sugary drinks. Thematic analysis resulted in the development of four themes relating to perceived prerequisites for effectiveness (perceptions of relevance, relatability), perceived aversiveness, opportunity for self-exemption and perceived potential to reduce sugary drinks consumption. Results of the analysis suggest the teaspoons labels were consistently considered by participants as strong performers across all themes. These labels provided new information, with no opportunity for consumers to discredit it. The information was considered relevant, ‘teaspoons’ were perceived as a measure that was relatable and provided an easy way to compare sugar content or to calculate total sugar consumption from beverages. Consistent with previous studies indicating that warnings on sugary drinks can elicit strong negative emotional reactions (e.g., worry, disgust), the information was perceived as aversive, with some participants expressing surprise and disgust [16]. Critically, most participants appeared to find it hard to self-exempt from this information.

The teaspoons labels appeared to prompt reconsideration of sugary drink consumption, first when they viewed the text-only version, and then again to a greater extent when they viewed the pictogram versions. This included perceptions that they would switch to a lower/no sugar option or decide not to consume the SSB. Participants perceived the quantity of sugar in a single drink as extreme and unnecessary, which they could readily understand through the text label “16 teaspoons of sugar”. However, adding the visual element of a teaspoon of sugar in the pictogram version appeared to strengthen the aversive response. This is consistent with other labelling research in SSB and tobacco showing that imagery can be more impactful than text only [36–39]. Further research using experimental methods is required to test how much more impactful the teaspoons image is compared to text only, and to test whether a similar effect would occur for other warning label categories.

All of the other labels that did not convey teaspoons of sugar, including the “high in sugar” pictogram, tended to have weaknesses in one or more themes. Some labels were perceived as having the potential to change knowledge (or at least were viewed as a useful reminder) but not behaviour. Some labels enabled self-exemption somewhat reminiscent of the way smokers may self-exempt from anti-smoking messages [40]; participants were regular sugary drink consumers who described consumption as enjoyable, and therefore were likely resistant to the idea of behaviour change in response to the information presented. It was of interest that labels that were found highly aversive by some participants (e.g., diabetes label stimulated thoughts of self-injection of insulin or exercise labels stimulated thoughts of effort required for exercise), participants could still self-exempt from these labels. These findings highlight that just because a label performed strongly on one theme, it may not necessarily be perceived by consumers to have real potential to motivate behaviour change. For example, a label may be perceived to be very useful, credible and relevant (prerequisites to effectiveness), but may still not be perceived by participants to motivate reduced consumption.

The concrete factual information presented in the teaspoons labels (both pictogram and text-only labels) made it difficult for participants to self-exempt. Teaspoons as a measure were easily understood. It is likely they require low health literacy to interpret the labels, which likely contributed to the ease of understanding that participants showed in response to this label. Labels could effectively communicate the high quantities of sugar (teaspoons) in common drinks. This is in contrast to the calorie and kilojoule disclosure labels, which were more abstract and notably required a high level of pre-existing knowledge, and in some instances were described as promoting consumption. These findings are consistent with previous qualitative findings relating to SSB warning labels, which suggest a preference for clear, concrete information, presented in everyday language, i.e., not in technical terms [20, 23]. Furthermore, a previous quantitative study indicated that displaying sugar content in cubes of sugar, also a concrete image, led to perceptions that a SSB was less appetizing compared to displaying the sugar content of an SSB in the more abstract measure of grams [41].

These findings align with principles from the health communication and warning label literature more broadly [42]. For a label to be effective, the threat must be perceived as credible. In general, health effects may or may not occur for consumers, and most often the effects are not instantaneous. Conversely, the amount of sugar consumed from a beverage is factual information, and the ingestion of multiple teaspoons of sugar is instantaneous for the consumer. Most participants found the idea of consuming high quantities of sugar immediately physically repulsive, and there was an underlying assumption that consuming that much sugar is unhealthy. While the illnesses conveyed on the health effects warning labels in this study are the main health consequences of consuming excess sugar, the health effects messages were not perceived to be as effective as the sugar teaspoons label. This further degree of separation from the consumption of sugar to the long-term consequences of health outcomes likely explains this finding; sugar ingestion is immediate, while the health effects were considered distant. The finding that health effects seemed distant is a common finding for studies involving young people [43]. Within the tobacco control literature, studies of young adults have also demonstrated that short-term risks (e.g., financial consequences or social risks) are typically more compelling than the longer-term health risks of smoking [44, 45]. It is of note that previous quantitative experimental studies have found labels warning of health effects led to greater reductions in hypothetical drink purchases than those depicting nutrient warnings [16]. However, the nutrient warnings used in the majority of these quantitative studies used “high in” or “excess” warning labels. The results of our study suggest that consumers may find the statement that a sugary drink is “high” in sugar difficult to contextualise, and it may be interpreted as an ambiguous message for sugary drinks. There was acknowledgement that most people already have some knowledge that these beverages are high in sugar; however, what constituted “high” or excessive was left to subjective interpretation. Consumers’ reactions to the teaspoons of sugar information may suggest they do not realise the excessive amount of sugar in beverages. This is supported by a national Australian study that found that over two thirds of the Australian adult participants underestimated or did not know the approximate amount of sugar (grams) within a typical soda or soft drink [8]. Providing information regarding a quantum of sugar in an easy to interpret FoP label for consumers (e.g., teaspoons, a common measure used in cooking and preparing food and drinks) may assist consumers in contextualising this information, and hence explain the strong reactions to the nutrient warning label used in this study. Further experimental studies to quantify these results will assist in determining whether labels depicting teaspoons of sugar lead to lower consumption intentions than other alternative warning labels, such as health effects or “high in” sugar labels.

Exercise equivalent information has been proposed as an alternative to conveying the amount of energy contained in foods and beverages due to the lower level of pre-existing knowledge required [36, 46]. In the current study, the exercise labels generated interest and discussion and had some perceived potential to change knowledge, but limited potential to reduce SSB consumption. Participants found ways to discount this information by interpreting it as falling within existing levels of daily activity, and not as additional exercise requirements. These results are consistent with findings of another study that indicated that while physical activity labels on FoP provide interesting information, the potential for behaviour change is limited [46]. This is in direct contrast to the American Beverage Association’s (and other industry lobby groups’) argument that improving knowledge regarding the balance of calories consumed and calories spent through exercise will alleviate the obesity and diabetes crisis [47].

A novel finding of this study is the potential of the energy warning label to be perceived positively with the risk that it may encourage increased consumption. “Energy” was not a clearly understood concept, and many participants perceived it to be beneficial, e.g., necessary to daily function and SSBs could provide a boost if energy levels are low. The potential for an “energy” label to increase consumption was exacerbated when reported with the low percentage of recommended daily intake (12%). This was perceived as providing permission to consume multiple beverages per day. This is of concern given that the energy label used in the current study was based on the information that is currently on beverage labels. A recent review of the Australian and New Zealand Health Star Rating system and sugar labelling has resulted in the removal of the energy icon-only option from November 2020, although industry has a 2-year transition period for implementation. These results are important for other countries using similar energy labelling.

In considering these results, it is important to acknowledge the limitations of this study, particularly in relation to generalisability of the findings. Participants were young Australian adult consumers who are already very familiar with public health strategies such as tobacco warning labels and tobacco control measures. As labels were shown in fixed order, there may have been a diminishing impact for labels shown later in the groups compared to those shown earlier in the groups. The labels were intentionally presented with minimal contextual information (such as size of the beverage) to encourage spontaneous and natural responses to the factual information that the labels provided. This approach allowed for natural discussion of when and how much contextualising of the label information was of importance to participants in their understanding and interpretation of the labels. However, we cannot be certain of consistency across participants in the precise type or size of drinks that they interpreted as relevant for the labels shown. Many participants’ initial reactions to labels (presented in a rectangular box, with ‘warning’) indicated recognition of similarities to tobacco warning labels in Australia, and facilitated initial perceptions of seriousness of the message and attenuation. Perceptions of the tested warning labels likely differ across countries, cultures, language groups and age groups, and design aspects of warning labels need further investigation. This qualitative study represents consumer perceptions and their understanding of labels. As noted, an experimental comparison of the effect of label types on consumption related behaviours (intentions, purchasing) would provide greater insight into whether these differences in label perceptions have the potential to translate into behavioural outcomes. MMresearch conducted the focus groups. While this company does not conduct market research for the beverage industry, participants were recruited from professional recruitment companies that may also conduct market research, and may also have clients from the beverage industry. These recruitment companies had no role in the design or implementation of this study. This risk is applicable to any studies that have recruited participants through online panels and market research participant pools, and participants of this study were screened regarding their employment or employment of close family/friends in the beverage industry. Finally, the groups conducted in the regional location were audio-recorded only and therefore, some visual cues that were apparent for visual recordings and viewing may have been missed in the analysis of these groups.

## Conclusions

This study provides important insights into consumer perceptions of a range of sugary drink warning labels. Of those presented, labels conveying the amount of sugar in a beverage in a factual, relatable and interpretable manner (via teaspoons) were perceived by consumers to have the greatest potential to impact consumption outcomes. Further experimental research would assist in quantifying these results and test the effectiveness of a simple but informative sugar labelling system.

## Supplementary Information

**Additional file 1: Supplementary material. **Discussion guide.

## Data Availability

Data not publicly available due to ethical and privacy restrictions. Data can be requested from the corresponding author (CM) with appropriate ethical approval.

## Abbreviations

FoP: Front of pack
SSB: Sugar-sweetened beverage
WHO: World Health Organization
US: United States of America
RDI: Recommended dietary intake
kJ: Kilojoules
