# Supplementary material for: “You can’t just eat 16 teaspoons of sugar so why would you drink 16 teaspoons’ worth of sugar?”: a qualitative study of young adults’ reactions to sugary drink warning labels
Source: BMC Public Health. 2022 Jun 22;22:1241. doi: 10.1186/s12889-022-13648-1 (PMC9219237; doi:10.1186/s12889-022-13648-1)
Supplement: Supplementary file 1 — Additional file 1: Supplementary material. Discussion guide. [file 12889_2022_13648_MOESM1_ESM.docx]

Supplementary material: Discussion guide

Purpose of the Discussion Guide

This discussion guide has been developed for the use of the group moderator to prompt discussion amongst the participants. This guide will be used to steer discussion to the topic areas to be covered, and the specific questions of interest within each topic area.

For the purposes of qualitative research, it is not necessarily intended that these questions be asked exactly as they are worded here. Focus group discussions are more like a conversation than a set of structured questions. The discussion will be as informal as possible and participants will be encouraged to speak openly and freely. The moderator will probe with questions such as “Can you tell me more about that?” and “What does that mean to you?” in order to understand participants’ responses. The moderator will make sure that all participants in the group have an opportunity to express their opinions.

Because each group of participants may be different, a responsive approach will be used for the research. Therefore, a level of flexibility will be taken in the conduct of each group to allow individual and group reactions to issues and to the advertisements. For this reason, the groups may vary in terms of the detailed topics and the order in which issues are discussed.

Explanation to participants:

- Introduce Group Moderator.
- Thank participants for time and contribution. Explain duration (1.5 hours).
- Explain refreshments, ask to turn phones off
- Explain what the research is about - “We’re here to talk about your thoughts on non-alcoholic beverages that contain sugar. All of the groups will be conducted with young adults who regularly drink these types of drink.”
- Explain recording, client viewing and confidentiality of participant information - “With your permission we would like to record the group. The recording will only be used to help us with analysing the results. Your personal details are confidential, and we will not keep or pass on any personal information about you. Is it OK for us to record the group?”
- If applicable, explain that the discussion will also be observed by researchers present in the adjoining room via a one-way mirror. They are there to learn from the groups in real time as it helps to develop the direction of discussion in subsequent groups. Reiterate that their personal details are confidential and that no personal information will be passed on.
- Explain the importance of honest opinions - “Your views and experience are important, so we would like you to tell us what you think and feel about your experiences and about each of the topics we talk about. There are no right or wrong answers to any of the issues we are discussing today, so it is important that you provide us with your honest opinions and that you understand that we will not make any judgements of you for your opinions. Also, as we are talking about your personal opinions and experiences, it is not necessary for everyone to agree with each other. It is helpful for us to find out the different opinions that people have, as well as where people have the same opinions, so please feel free to tell us whatever you think and feel, even if it might be different to what other people in the room are saying. It is also important that you know that everything that we talk about today is treated with confidence, and we expect that you will also treat anything that you hear in the discussion with confidence, thanks.”

Introductions

I’d like to start by asking a bit about yourself: your first name, where you live, if you work full time/part time or are studying.

**SSB Consumption**

Because we are talking about beverages today, what I would like to do first is get a bit of a sense of what beverages everyone drinks. Can we go around the table and can you tell me what the normal pattern is for the day / week of what you would drink.

(moderator to keep list on white board / sheet of all beverages that people consume).

Context around consumption

- What are the main reasons for consuming these drinks?
  - If needed, probe for associations with food, alternative to alcohol, source of energy
- Where do you usually drink these beverages?
  - If needed, probe for whether consumed alone or with others, at home, work, leisure
- In general, what are the benefits of drinking these types of beverages?
- Are there any reasons why these beverages should not be consumed?

Perceptions about consumption

- Do those around you drink about the same amount as you or is it higher or lower?
  - If different, how does that make you feel about your own level of consumption?
- When you think about the beverages you consume, how do you feel?
- Do you feel that is ok from a health point of view?
- Would you prefer to have more / less of any of those? Which ones? Can you tell me more about that?

**SSB Knowledge**

- And thinking about all of those drinks, which ones would you say are sugar sweetened?
- Which ones are sweetened by something other than sugar?
  - What sweeteners are used in these drinks?
- Which ones are unsweetened?
- How can people tell the difference?
  - How do you tell the difference?
    - Is this something that you just know, or do you look on the Nutrition Panel?
    - Are you interested in knowing whether / how much sugar is in a drink?
- Just looking at the sugar sweetened drinks, and assuming a standard volume, I’d like the group to rank these drinks from the drink that you think has the most sugar to the one with the least.

**SSB Preferences**

- And thinking about the sugar sweetened drinks, what is it that you like about them?
  - How do they compare with drinks that are sweetened some other way? What is the difference?
  - How do they compare with unsweetened drinks? Water?
  - Probe whether taste, or other differences?
- When / why do you choose to drink SSBs?
- When / why would you choose a SSB over a non-SSB at a particular time?
- Other than taste, what is the main difference in your experience / consideration of those drinks?
- How do you feel about the amount of SSBs that you drink? Can you tell me more about that?
  - What would you like to be different? Can you tell me more about that?
  - Why do you have more / less SSB than you would like?

Moderator note: The Visual stimuli can be introduced at various points throughout the remainder of the discussion to prompt for further insight into the topics

**Health**

- What do you know about the health benefits associated with consuming sugar sweetened i) soft drinks, ii) sports drinks or iii) energy drinks?
- What do you know about the health problems associated with consuming sugar sweetened i) soft drinks, ii) sports drinks or iii) energy drinks?
  - How do you feel about these stories of the health problems?
  - Do you believe them? Can you tell me more about that?
- Have you heard any recommendations about limiting intake of SSBs?
  - How do you feel about these recommendations?
  - Are you likely to follow them? Can you tell me more about that?

**Advocacy**

- Evidence shows that consuming sugar sweetened drinks such as soft drinks contributes to obesity in adults and children and so some health groups want to regulate consumption…
  - What, if anything, do you think needs to be done
  - What responsibility do you think the government has in this area? Why do you say that?
  - What do you think they could do?
  - What would have to change for you to think that there was a need for SSBs to be regulated?
    - prompt re knowledge of health effects?
    - other knowledge?
- One way of reducing consumption may be to include a tax incorporated as a price increase on soft drinks.
  - What are your thoughts on this?
  - Would you support a tax? Can you tell me more about that?
  - How would you feel about a government taking such a measure?
- Another measure is to add warnings to the packaging of drinks that contain high amounts of sugar.
  - What are your thoughts on this?
  - Would you support such requirements? Can you tell me more about that?
  - How would you feel about a government taking such a measure?

**Warning statements** (Moderator note: when warning statements are introduced, use the following probes to elicit responses about each label and comparisons between labels)

- Have a look at the warning statements and tell me your thoughts about them
  - In what way does the label grab your attention? What stands out?
  - Which labels do you think are ‘powerful’?
    - Teach you something new
    - Convey believability
    - Make you stop and think
    - Are relevant to you
    - Make you feel uncomfortable about drinking sugary drinks
    - Make you feel uncomfortable about your current sugary drink consumption
